# The Life Cycle Assessment for Polylactic Acid (PLA) to Make It a Low-Carbon Material

**DOI:** 10.3390/polym13111854

**Published:** 2021-06-02

**Authors:** Erfan Rezvani Ghomi, Fatemeh Khosravi, Ali Saedi Ardahaei, Yunqian Dai, Rasoul Esmaeely Neisiany, Firoozeh Foroughi, Min Wu, Oisik Das, Seeram Ramakrishna

**Affiliations:** 1 Center for Nanotechnology and Sustainability, Department of Mechanical Engineering, National University of Singapore, Singapore 117581, Singapore; fatemeh_khosravi22@yahoo.com; 2 Department of Polymer Engineering, Faculty of Engineering, Golestan University, P.O. Box 491888369, Gorgan 1575949138, Iran; Alisaedi2012@yahoo.com; 3 School of Chemistry and Chemical Engineering, Southeast University, Nanjing 211189, China; wuminnj@163.com; 4 Department of Materials and Polymer Engineering, Faculty of Engineering, Hakim Sabzevari University, Sabzevar 9617976487, Iran; r.esmaeely@hsu.ac.ir; 5 Department of Materials Science and Engineering, Faculty of Engineering, National University of Singapore, 9 Engineering Drive 1, Singapore 117576, Singapore; firoozehforoughi@u.nus.edu; 6 Department of Civil, Environmental and Natural Resources Engineering, Structural and Fire Engineering Division, Luleå University of Technology, 97187 Luleå, Sweden; oisik.das@ltu.se

**Keywords:** polylactic acid, greenhouse gas, life cycle assessment, carbon dioxide, low carbon

## Abstract

The massive plastic production worldwide leads to a global concern for the pollution made by the plastic wastes and the environmental issues associated with them. One of the best solutions is replacing the fossil-based plastics with bioplastics. Bioplastics such as polylactic acid (PLA) are biodegradable materials with less greenhouse gas (GHG) emissions. PLA is a biopolymer produced from natural resources with good mechanical and chemical properties, therefore, it is used widely in packaging, agriculture, and biomedical industries. PLA products mostly end up in landfills or composting. In this review paper, the existing life cycle assessments (LCA) for PLA were comprehensively reviewed and classified. According to the LCAs, the energy and materials used in the whole life cycle of PLA were reported. Finally, the GHG emissions of PLA in each stage of its life cycle, including feedstock acquisition and conversion, manufacturing of PLA products, the PLA applications, and the end of life (EoL) options, were described. The most energy-intensive stage in the life cycle of PLA is its conversion. By optimizing the conversion process of PLA, it is possible to make it a low-carbon material with less dependence on energy sources.

## 1. Introduction

Nowadays, plastics are employed widely in different industries, such as construction, packaging, electronics, clothing, healthcare, and so on, due to their excellent physical, chemical, and mechanical properties, and economic viabilities compared to traditional materials [1,2,3,4]. The global plastics manufacturing started from 1.5 million tons in 1950, and reached 322 million tons in 2017, and is predicted to increase to 1.63 billion tons in 2050 [5,6]. This huge amount of plastics production worldwide has made its disposal a considerable global concern, with a great potential to harm the environment, humans, and animals. Plastics are found in seawater, jungles, or municipal solid wastes. More than 8.3 billion tons of plastics were produced in the span of 1950 to 2015, in which less than 20% were recycled or incinerated, and the rest were left in the environment or were landfilled [7,8]. The environmental issues and ecological impact associated with plastics have led to more studies and research into developing more sustainable materials. Currently, new factors such as recyclability and biodegradability are taken into account when developing new plastics [2,9]. Despite the highly developed circular economy, plastics are still being observed in the environment [10]. Hence, it can be concluded that biodegradability may be the most important factor to address the environmental aspects of plastics [11]. 

Bioplastics or biodegradable polymers are the potential candidates to replace fossil-based plastics due to using renewable resources and significantly less greenhouse gas emissions (GHE) [12,13,14]. Bioplastics are fully or partially derived from bio-based and renewable origins such as agriculture or marine products, which can help in CO_2_ absorption during their production process [15,16]. The absorbed carbon will finally be released when the life span of the product is over [17]. This is how these bio-based plastics avoid consuming additional fossil-fuels as feedstock [18]. However, the production of bioplastics is still dependent on fossil fuels as the source of energy in their fabrication process, which can also be eliminated in the future through using renewable resources. Due to the developing market share of bioplastics, it is important to increase our knowledge on the economic and environmental aspects of bioplastics. LCA is a tool that provides quantitative information about environmental sustainability, or “cradle to grave” of a bioplastic [15,19,20,21].

PLA is considered as one of the most prevalent and commercial bioplastics worldwide, with a production of 0.2 million tons in 2015 and 0.3 million tons in 2019. PLA is fabricated from lactic acid, which is produced from the fermentation of the starch present in sugarcane and corn [7]. PLA is used in different industries, such as healthcare, textile, packaging, and so on [22,23,24]. Historically, the biomedical applications of PLA date back to the 1970s, when it was used as sutures [25]. Afterward, it gained considerable attention in the 1980s from Cargill, Dupont, and Coors Brewing, and then it was produced on large scales. The majority of the manufactured PLA is employed in packaging [26]. Furthermore, due to the biodegradability of PLA, it provides several EoL options, including mechanical recycling, chemical recycling, landfilling, and industrially composting [27,28]. It should also be noted that compostability is the same as biodegradability but under aerobic conditions for 6–12 weeks [29,30]. The EoL options help with the circularity of PLA and managing for a circular economy. In this regard, this paper aims to provide an LCA for PLA to help manufacturers and consumers with proper sustainable approaches in the life span of PLA, including usage, manufacturing, and disposal. First, the life cycle of PLA is discussed in four main stages, including feedstock collection and its conversion, processing, applications, and EoL options. Second, a comprehensive literature review of the existing LCAs for PLA is presented. Finally, we come up with specific suggestions to make PLA a low-carbon material by exploiting the available GHG emissions data. 

## 2. The Life Cycle of PLA 

**Figure 1:**
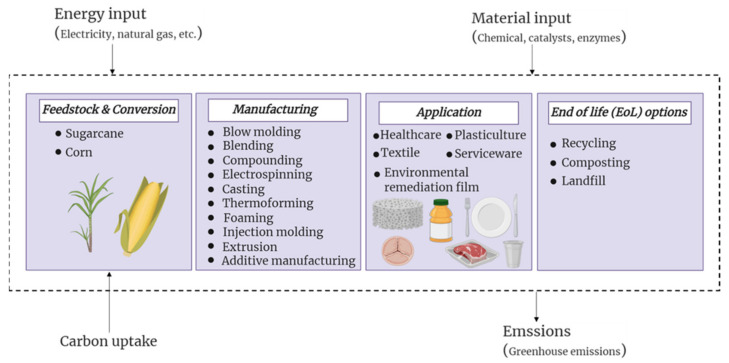
PLA life cycle with potential emissions at different stages.

The lifecycle of PLA is discussed through its waste management scenarios to determine the main drivers influencing its environmental aspects. Figure 1 depicts the system boundary of PLA production direction regarding energy, materials, and emissions flow. The pathway is divided into five stages, including (1) feedstock collection and its conversion, (2) processing, (3) use, and (4) EoL.

### 2.1. PLA Feedstock Collection and Its Conversion

The first step of the lifecycle of PLA is feedstock collection and conversion, according to Figure 1. PLA manufacturing consists of three main steps. Firstly, the bio-based sources such as corn or sugarcane should be collected and transported to a plant. Then, the feedstock is converted to lactic acid through the fermentation process of starch or sugar. This is the most common manufacturing method of lactic acid as it is chemical- and cost-efficient and leads to the fabrication of pure lactic acid. One of the main factors affecting the crystallinity and biological degradation of PLA is the optical purity of the lactic acid, and hence even a low concentration of impurities matters. Therefore, enough attention should be paid to the downstream processing of lactic acid, as the fermentation broth contains considerable amounts of impurities. The identification and separation of the impurities is a crucial step that determines the final properties of PLA. Finally, the as-synthesized lactide, the dimer of lactic acid, is polymerized to produce PLA via ring-opening polymerization. Due to the chiral characteristic of lactic acid, there are three forms of lactide, including L-lactide, D-lactide, and D, L-lactide. The production of optically pure high molecular weight PLA significantly depends on the line stream monitoring in the whole process.

**Figure 2:**
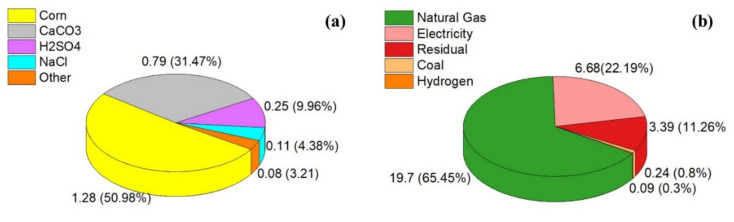
The (**a**) materials in kg and (**b**) energy in MJ used for one kilogram of PLA production.

The amount of different materials and energy used along the pathway of producing one kilogram of PLA from corn is summarized in Figure 2 [2]. According to Figure 2, natural gas and electricity take up most of the total energy used in the process, with 65% and 22%, respectively. It should be noted that there are some other materials or energy parameters that were not included in Figure 2 due to their negligible role.

### 2.2. PLA Processing

The second step toward the life cycle of PLA is the manufacturing of PLA products. It is worth mentioning the physicochemical and mechanical properties of PLA in this section to help with a better understanding of its applicable manufacturing methods. PLA is one of the biodegradable thermoplastics, with similar properties to polystyrene (PS) and polyethylene terephthalate (PET) [31,32]. Table 1 summarizes the physical and mechanical properties of PLA. As can be seen in Table 1, there is a range for each property due to different types of PLA isomers, different used natural sources, and different production procedures [33].

Based on the thermal history and stereochemistry of PLA, in the solid-state, there are both types, semi-crystalline and amorphous [34]. Regarding semi-crystalline PLAs, both Tm and T_g_ are important values to predict its behavior for different applications [35]. On the other hand, transmission from a glassy structure to rubbery can occur above T_g_ for transparent amorphous PLAs [36]. Below T_g_, the glassy structure of PLA with creep behavior will be formed until cooling to its β-transition at 60 °C. In addition, there are numerous solutions for PLA products, such as dioxane, acetonitrile, chloroform, methylene chloride, 1,1,2-trichloroethane, and dichloroacetic acid [37,38,39]. At boiling temperature, PLAs are soluble in ethylbenzene, toluene, acetone, and tetrahydrofuran. It should be noted that PLA is insoluble in water. Polymers based on lactic acid have a wide range of mechanical properties, varied from elastic plastics to high-strength polymers, depending on the semi-crystalline and amorphous structure and the degree of crystallinity. 

Semi-crystalline PLAs indicate higher mechanical properties compared to amorphous PLAs. The mechanical properties of PLA are strongly affected by molecular weight (Mw), as by increasing the Mw from 50 to 100 kDa, the tensile strength and modulus of PLA were doubled [40]. Furthermore, controlling the stereochemical architecture of polymers based on lactic acid by polymerization with L-lactide, D-lactide, D, L-lactide, and meso-lactide leads to control the rate and speed of crystallinity, which have a significant impact on the quality of mechanical properties [41,42,43].

Considering the above-mentioned properties of PLA, there is a wide range of applicable manufacturing methods for PLA products in the forms of fibers, films, parts, and so on. PLA products can be fabricated on a large scale using blow molding, blending, compounding, electrospinning, injection molding, casting, thermoforming, foaming, extrusion, and additive manufacturing [33]. Among all the applicable manufacturing methods for PLA, extrusion and injection molding are the most used [52]. There is energy balance information for both manufacturing methods for manufacturing one kilogram of plastic, including PLA, according to Keoleian et al.’s study [53]. The extrusion-made PLA products use 2 MJ/kg electricity with 1.01 mass input factor (MIF), whereas injection molded ones use 7.2 MJ/kg with 0.95 MIF.

### 2.3. Photodegradation of the Samples

**Figure 3:**
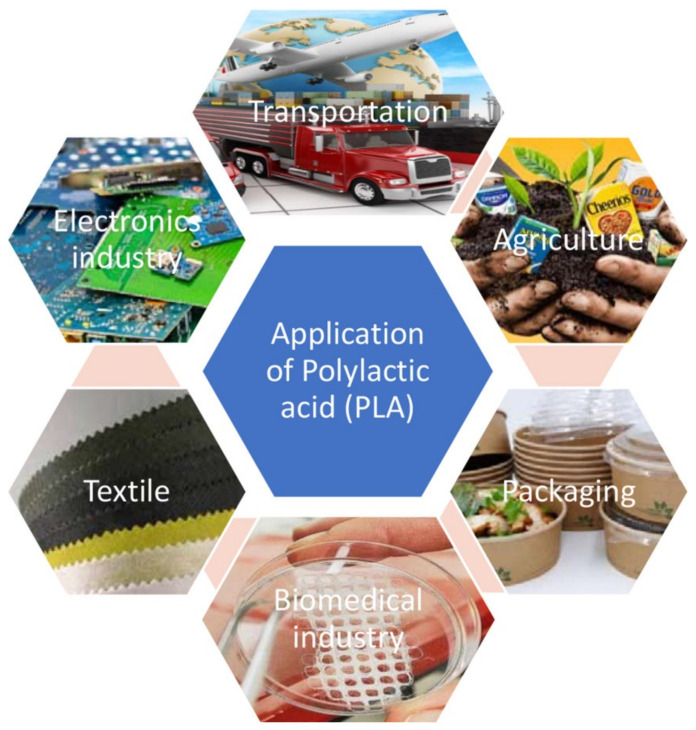
PLA applications [55].

The third step in the life cycle of PLA is its use. Therefore, the applications of PLA are discussed here, as shown in Figure 3. PLA was firstly used for only medical purposes as it was rare and expensive. At present, since the availability of high molecular weight PLA, its products can be fabricated via all the aforementioned manufacturing methods, especially extrusion and injection molding. Owing to the comparable properties of PLA with PS and PET, PLA covers a wide range of applications [54].

The major use of PLA is in the packaging industry [56,57]. PLA use is rapidly growing as “green” food packaging, which is widely considered in the fresh products field and has become the best option for fruit, vegetables, and salad containers in retail markets [58,59,60]. On the other hand, delicatessen and fast-food restaurants use disposable cutlery, drinking and salad cups, plates, and containers which are manufactured by biodegradable polymers for serving foods [24]. These types of productions are in contact with various acidic and high-cholesterol foods with different storage temperatures, varying between below 25 and above 60 °C. Thus, the mechanical, physical, and optical properties of PLA must be tailored according to the packaging applications. 

PLA has been widely used in various biomedical applications such as stents, plates, and screws for craniomaxillofacial bone fixation, interference screws in the ankle, spinal cages, soft-tissue implants, tissue engineering scaffolds, tissue cultures, and drug delivery devices, due to its biocompatibility [61,62]. PLA is considerably utilized in vascular stent applications due to its bioabsorbable property and favorable degradation behavior, and has the potential to be an appropriate replacement for metallic stents. Among PLA isomers, poly (L-lactic acid) (PLLA) is the most common biopolymer used for stent applications. PLA is a potential candidate for drug delivery systems due to its wide drug-releasing options. The PLA drug release occurs in several steps, including the breakage of ester bonds through hydrolytic cleavage, the transformation of hydrolytic products into non-toxic sub-products, exiting non-toxic products through natural cellular activities, and urine. In addition to the above applications, nanoparticles of PLA are used to encapsulate various drugs such as restenosis, oridonin, and so on. In addition, PLA is used for orthopedic devices. The most important reason for using biopolymers, e.g., PLA rather than metallic structures, is avoiding second surgery to remove the orthopedic devices, which reduces the costs and makes for a more facile recovery. The main uses of PLA in orthopedic devices are screws, fixation pins, plates, and suture anchor. Another application of PLA in the biomedical section is for tissue engineering (TE). One of the most applicable PLA forms in TE is three-dimensional (3D) porous scaffolds used for cell culturing applications, such as cardiovascular diseases [63]. The 3D PLA structure is affordable by electrospinning and 3D printing for patient-customized products [64]. In addition, PLA is also used in textile, plasticulture, service-ware, and environmental remediation films [52].

### 2.4. PLA EoL Options

**Figure 4:**
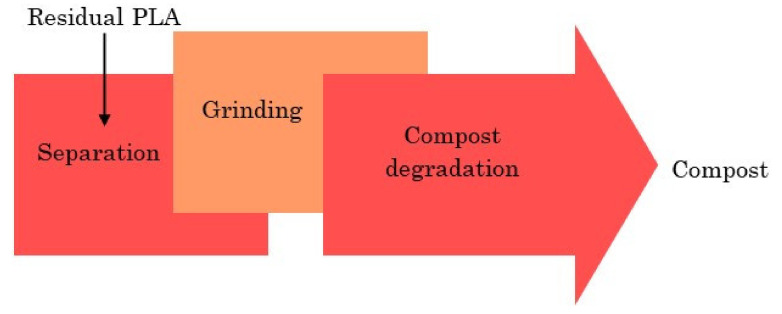
PLA composting procedure.

Commonly, the EoL options include landfilling, composting, anaerobic digestion, incineration or thermal treatment, and recycling [65]. In this paper, the most common EoL options for PLA are considered, including landfilling, composting, and recycling. PLA and its products are biodegradable, but it does not allow for littering them in the environment or self-composting. PLA products are still stable in soil and their landfilling merely affects the environment because only one percent will be degraded after 100 years [66]. PLA, as a promising synthesis biopolymer, is difficult to degrade at natural environment temperature. The inherent slow crystallization kinetics of PLA lead to the slow degradation rate of PLA which occurs under anaerobic thermal conditions [67,68]. The CH_4_ production of PLA landfilling at ambient temperature is below 0.1%, with insignificant CO_2_ emissions. Although PLA products are generally produced for a short life span, their reuse is also achievable. Composting is generally considered as one of the worst EoL options due to no energy recovery and low compost quality. Figure 4 illustrates the different stages of PLA composting. The PLA composting process includes three steps, separation, grinding, and compost degradation. The organic compound, or the source of the nutrients, is the result of the composting process. It is noticeable that the energy resources have not been used during the composting process [69].

**Figure 5:**
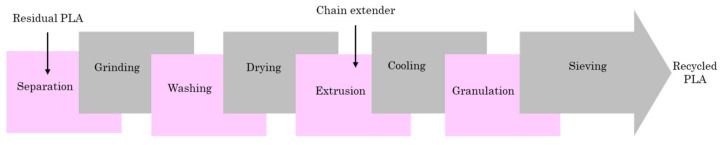
PLA mechanical recycling procedure.

In addition, mechanical and chemical recycling are also two EoL options for PLA [70]. By avoiding using virgin PLA, a significant reduction in GHE and environmental issues is witnessed. Figure 5 presents the mechanical recycling of PLA. According to Figure 5, the process of PLA mechanical recycling contains eight main steps, including separation, grinding, washing, drying, extrusion, cooling, granulation, and sieving. The incorporation of a chain extender during the extrusion process can enhance the mechanical properties of the recycled PLA [71,72,73]. 

**Figure 6:**
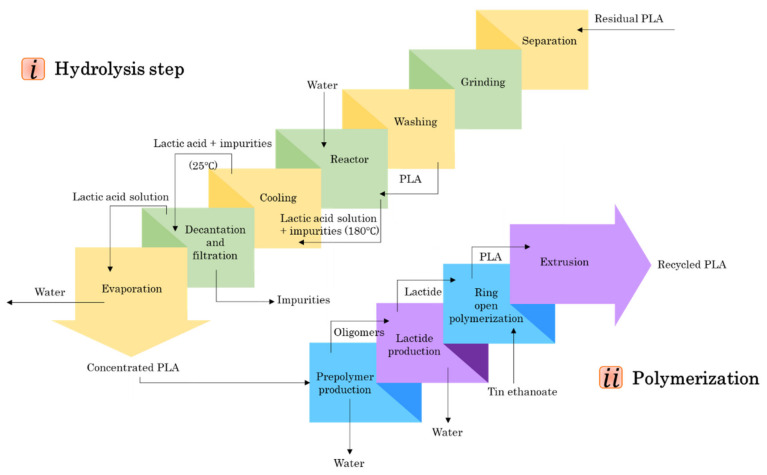
PLA chemical recycling procedure.

The chemical recycling process is categorized into hydrolysis and polymerization stages. As can be observed in Figure 6, the hydrolysis stage consists of separation, grinding, washing, reactor sector, cooling, decantation and filtration, and evaporation. Based on Marina et al.’s [69] study, the reactor ought to be immersed in insulating oil and the process temperature remained unchanged at 180 °C for 2 h. Moreover, the impurities added before the cooling step were removed in the decantation and filtration step. Ultimately, the concentrated lactic acid is produced by water evaporation for polymerization. The four main steps in polymerization are prepolymer production, lactide production, ring-opening polymerization, and extrusion. 

Although there are considerable attempts to develop recycling of the materials, PLA recycling is limited due to the poor available infrastructures. Considering the high cost of separation and the poor quality of the recycled PLA, the only applicable recycling method for PLA is “mixed” recycling [52]. Hence, the most plausible EoL option for PLA is landfilling. As mentioned earlier, PLA is biodegradable and will be degraded to H_2_O, CH_4_, and CO_2_. In other words, PLA biodegradation participates in the total amount of GHE in the life cycle of PLA [74].

## 3. Summary of the Existing LCAs of PLA

A summary of the existing life cycle studies on PLA and its products was extracted from the literature and is presented in Table 2. Comparing the characteristics, objectives, assumptions, data sources, and major findings of these studies will provide insights into better PLA LCA and address the environmental issues. 

One of the pioneering studies on LCA of PLA dates back to 2003, on the NatureWorks™ PLA [75]. According to this study, the total required fossil energy for PLA was less than fossil-based polymers which can be used in other sections of the PLA production procedure. Later, in 2009, Madival et al. investigated the LCA of PLA clamshell containers in comparison with PET and PS clamshell containers [76]. According to the results, the PLA containers could be 100% recyclable and/or compostable. Moreover, PLA had less GHE (~28 kg CO_2_) compared to PET (~830 kg CO_2_) distributed by 16-ton trucks [76]. Then, Piemonte examined the PLA total energy demand and environmental impact in comparison with PE and PET in 2011 [77]. They found that bio-plastics usage instead of fossil-based plastics can lead to considerable energy and GHE savings [77]. Subsequently, in 2014, Papong et al. carried out a comparative investigation on the environmental impact of PET and PLA drinking water bottles from a life cycle outlook. The results showed that the production of PLA bottles can lead to a reduction in CO_2_ emissions, lower toxicity, and less demand for non-renewable energy [78,79]. In the same year, Mahalle et al. studied a cradle-to-gate LCA of polylactic acid/thermoplastic starch (PLA/TPS) and wood fiber-reinforced PLA bio-composites [80]. According to the results, bio-composites are able to perform in a more environmentally friendly manner in comparison with PP [80]. In 2015, Benetto et al. examined the LCA of PLA and TPS multilayer film designed by atmospheric plasma usage. Two system boundaries and two EoL were carried out, namely, cut-off, expansion, recycling, and incineration, respectively. Cut-off had a higher impact in comparison with expansion. In disposal, incineration and recycling had negative values for one kg of multilayer in I2002 [81]. Later, in 2017, Hottle et al. investigated the production of biopolymers and EoL comparisons through LCA [13]. Based on the results, recycling is able to reduce 40% to 60% of environmental impacts in fossil fuel depletion for petrochemical polymers [13]. In addition, Maga et al. studied the LCA of the PLA and its recycling options in 2019 [70]. They examined mechanical, solvent-based, and chemical recycling of the waste PLA. Based on the results, recycling PLA led to higher savings (0.3–1.2 times higher) in GHE compared to the PLA incineration. Furthermore, recycling had less cumulative energy demand (CED) compared to incineration [70]. In the same year, Morão et al. investigated the PLA’s life cycle impact (LCI) produced by sugarcane in Thailand [15]. According to the results, several approaches were introduced to improve the PLA environmental impact, such as enhancement in the farming practice of sugarcane, exploitation of bagasse boilers with higher efficiencies at sugarmill, consumption reduction in auxiliary chemicals, and renewable energy usage enhancement in the sugar conversion process to PLA [15]. One of the most recent available LCAs of PLA was conducted by Bałdowska-Witos et al. for the PLA bottle shaping’s environmental impact assessment in 2020 [82]. The results demonstrated that the GHE in the environment was affected by water, electrical energy, and raw materials usage during the bottle shaping process [82]. 

## 4. Summary of the Existing LCAs of PLA

**Figure 7:**
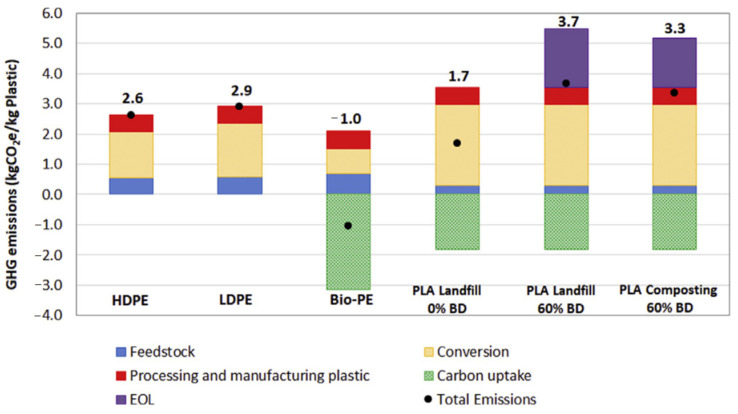
GHE balance in the life cycle of PLA products with different EoL options compared with different PE grades [2].

Investigation of the GHE in the life cycle of a material can help with the best suggestions for making it a low-carbon material. As mentioned in the previous sections, most of the PLA products end up in landfilling or composting. In this section, the CO_2_ emission of PLA in three different EoL options including landfilling without biodegradation and landfilling or composting with 60% biodegradation is evaluated. The PLA CO_2_ emission is compared with PE products. GHE of all these materials is summarized in Figure 7. 

As PE is not considered as a biodegradable material, there are no EoL emissions displayed for it in Figure 7. It can be observed in Figure 7 that carbon uptake is considered only for biopolymers, which is their advantage in terms of environmental aspects compared to fossil-based plastics [83]. One kg of PLA is calculated to be able to uptake around 1.8 kg of CO_2_. Regarding the total GHE of PLA landfill with no biodegradation, it can be concluded that it releases 1.2 and 0.9 kg of CO_2_ per kg of PLA less than LDPE and HDPE, respectively. Opposed to this, in the cases that the biodegradability of PLA is taken into account, the total GHE of PLA will enhance greatly, more so than HDPE and LDPE.

It should be noted that PLA is in the early steps of its progress and its production and conversion processes are not optimized compared to PE, which owns the first rank in terms of production worldwide among plastics [84]. By optimizing the conversion process of PLA, it is possible to reduce the energy demand and GHE of the procedure. For example, NatureWorks has been producing PLA for more than 15 years and is optimizing the processing of PLA. Therefore, it seems that one of the best suggestions for making PLA a low-carbon material is optimizing its conversion process, as it consists of more than 50% of PLA GHE in both landfilling and composting. In fact, PLA conversion releases about 2.9 kg of CO_2_ per kg of PLA. The NatureWorks optimization shows that they were able to develop the production of PLA and could reach only 0.6 kg of CO_2_ emission per kg of PLA. However, that data is not available to the public. This clearly shows the high potential of optimization of the PLA processing in reducing the GHE and coming up with more environmentally friendly PLA. Another suggestion to make PLA a low-carbon material is to develop recycling facilities to obtain new PLA products from the recycled PLA, of good quality and acceptable properties. By recycling, the EoL emissions, which are of considerable amounts, will be removed from the calculations. 

## 5. Conclusions

Unlike fossil-based polymers, bio-based polymers derived from renewable origins offer more CO_2_ absorption during their production process. However, the production of bio-based polymers is still dependent on fossil fuels as the source of energy in their fabrication process. PLA is considered as one of the most prevalent and commercial bio-based polymers for numerous applications, with several EoL options, including mechanical recycling, chemical recycling, landfilling, and industrial composting. However, when the lifetime of PLA-based products is over, they will be mostly landfilled or composted. The lack of proper infrastructures for PLA processing leads to limitations to recycling them. There are several LCAs of PLA or comparing different plastics with PLA in terms of environmental aspects, energy demand, and GHE. By exploiting the LCAs of PLA, it can be optimized to be a more environmentally friendly material. The GHE attributed to the life cycle of PLA shows that the conversion of the bio-sources to lactic acid and then PLA is an energy-intensive process that releases a huge amount of CO_2_ to the atmosphere. According to the available data, more than 50% (2.8 kg CO_2_/kg PLA) of the released CO_2_ in the PLA life cycle belongs to its conversion. By optimizing the conversion process of PLA, there will be a high potential to make PLA a low-carbon material. 

## Figures and Tables

**Table 1:** The physical and mechanical properties of PLA. Table summarized based on data from Refs. [44,45,46,47,48,49,50,51].

Properties	PLA
Polymer density (g/cm^3^)	1.21–1.30
Tensile strength (MPa)	15.5–150
Tensile modulus (GPa)	2.7–16
Ultimate strain (%)	2–10
Specific tensile strength (Nm/g)	16.8–66.8
Specific tensile modulus (kNm/g)	0.28–3.85
Glass transition temperature (°C)	60–65
Melting temperature (°C)	130–180

**Table 2:** The summary of the available LCAs on PLA.

Subject	Goal and Scope	LCA Software/LCIA Methodology	Key Assumptions	Data Sources	Major Findings	Ref.
PLA manufacturing/Raw materials	Cradle-to-grave LCA of PLA production	Based on Association of Plastics Manufacturers of Europe (APME) analysis	-	Based on APME, LCI databases	(1) The production processes of PLA are capable of being both sources of carbon credit and fossil-energy-free (2) Being lower in fossil energy use and greenhouse gas emissions compared to conventional polymers based on petrochemicals (3) Major impact: climate change	[75]
PLA manufacturing/Raw materials/EoL	Cradle-to-cradle LCA of PLA compared to PET and PS thermoformed clamshell containers and consideration of their environmental impacts based on different LOI scenarios	SimaPro^TM^/Eco-Indicator	(1) All three types of containers have the same mold (2) The filling operation of each type of container is excluded (3) Total amount of waste: PET = 3.61%, PS = 3.15%, PLA = 3.19% (4) Composting as an EoL scenario is not considered	Ecoinvent databases available with SimaPro^TM^, Commercial LCI databases	(1) The PLA containers are capable of being 100% compostable and/or recyclable (2) Major impact: global warming, aquatic ecotoxicity burdens, and ozone layer depletion affected by transportation stage of polymers	[76]
Recycling and manufacturing of PLA, LOI	Investigating the LCA of PLA for three different recycling technologies for post-consumer and post-industrial waste to identify their environmental impacts compared to thermal treatment	GaBi software/Institute for Energy and Environmental Research, Heidelberg GmbH ifeu	(1) Enough PLA in the lightweight packaging (LWP) waste stream (2) Transmission of 100% PLA fraction from the waste to the thermal treatment (3) Thermal treatment as the reference EoL option	Lab and pilot plant data, Commercial LCI databases	(1) Superior savings (0.3–1.2 times higher) in GHG emissions when utilizing PLA recyclates compared to incineration (2) Having a lower CED of recycling in comparison with waste incineration (3) PIW and PCW lead to energy recovery in case of heat and electricity	[70]
PLA manufacturing/Raw materials	Cradle-to-gate LCA of PLA production from sugarcane in Thailand considering its environmental impacts	SimaPro 8.4./Cumulative Energy Demand (CED)	(1) The toxicity is excluded from environmental impact categories	Commercial LCI databases	(1) Major impacts: Global warming potential, eutrophication, water, particulate matter, land use, acidification (2) Considerable improvement measures in PLA’s environmental impact reduction: enhancement in the farming practices of sugarcane, better yield bagasse boilers at the sugarmill, increase in the renewable energy usage in the conversion process, and reducing the assistant chemicals’ usage	[15]
PLA manufacturing/PLA products	Cradle-to-grave LCA of PLA bottle shaping and its environmental impacts identification	SimaPro 8.4./eco-indicator-99 (Damage Level)	(1) Beverage bottling, labeling, storage, and distribution were excluded from the production process (2) Storage and transportation of raw materials were excluded	-	(1) Emission of nitric oxides, carbon dioxides, and sulfur oxides into the natural environment affected by electrical energy, water, and raw materials utilization during the bottle shaping process (2) End product degasification and cooling have the most important role in the emissions and fine particles’ formation (3) Major impacts: global warming, water resources’ usage, fine particles’ formation, water acidification, and land use	[82]
PLA manufacturing/PLA products	Cradle-to-gate and cradle-to-grave LCA of PLA and Mater-Bi	SimaPro7.2/Cumulative Energy Demand (CED), EI-99	(1) The average transportation distance of PLA and Mater-Bi products = 100 km (2) Biodegradation degree of PLA and Mater-Bi in the anaerobic digestion process = 85% (3) Mechanical recycling based on two options: open-loop LCA and closed loop LCA	Ecoinvent v.2.2 database	(1) Utilization of bioplastics instead of conventional plastics leads to significant GHGs emissions and energy savings (2) Energy consumption of PLA compared to PE and PET is 50% from fossil resources (non-renewable)	[77]
PLA manufacturing/Raw materials/EoL	Cradle-to-gate LCA of PLA drinking water bottles compared to PET bottles	SimaPro/CML 2 baseline 2000	(1) The CO_2_ required for photosynthesis from the solar energy and air is excluded (2) Out of total applied nitrogen fertilizer, 1% evaporated as N_2_O-N and 10% as NH_3_ (3) Efficiency of electricity production = 30%	Literature, calculations, Ecoinvent database, IPCC method, Commercial LCI databases	(1) Reduction in non-renewable energy demand, CO_2_ emissions, and human toxicity by PLA bottles production (2) High GHG emission induced by cassava-based PLA resin compared to corn- and sugarcane-based PLA (3) Major impacts: landfill, incineration, recycling, and composting	[78]
PLA/TPS manufacturing/Raw materials	Cradle-to-gate LCA of wood fiber-reinforced PLA and PLA/TPS bio-composites in comparison with PP	None/Cumulative Energy Demand (CED)/TRACI	(1) The flows that contained less than 1% of the cumulative mass might be excluded (2) The flows that contained less than 1% of the cumulative energy might be excluded	US LCI database, US-EI database	(1) Major impacts: global warming, land and water acidification, stratospheric ozone depletion (2) TPS is less effective in environmental impacts than PLA (3) Better performance in terms of environmental issue belonging to bio-composites compared to PP, except for eutrophication effects if manufactured utilizing hydroelectricity	[80]
PLA manufacturing/Raw materials/EOL	Cradle-to-grave LCA of PLA and TPS multilayer film	SimaPro 7.3.3/Impact 2002+(I2002), ReCiPe	(1) Stiffness has a linear relationship with elasticity (2) The amount of energy according to the environmental data is replaceable with conventional productions based on a system expansion approach (3) Biodiversity and water usage are excluded	Ecoinvent 2.1 database, Lab and pilot plant data	(1) From two system boundaries that are followed: cut-off possesses a higher impact in comparison with expansion (2) Incineration and recycling possess negative values in the disposal’s Damage assessment for one kg of ML in I2002	[81]
Bio-based polymers and traditional plastics/manufacturing/EoL	Cradle-to-grave LCA of bio-based polymers and traditional plastics followed by EoL investigation	None/TRACI	(1) The plastics’ utilization and formation of the product were excluded (2) LDPE was considered as film waste and modeled like the MRF (material recovery facilities) process scenarios	Literature sources	(1) Gaining 100% damage level for petrochemical polymers’ production impact in impact categories (2) Highest global warming induced by TPS and PLA landfilling (3) Recycling can reduce environmental impacts by 40% to 60% in fossil fuel depletion for petrochemical polymers	[13]

## Data Availability

The data presented in this study are available upon request from the corresponding author.

## References

[1] Vijay Kumar V., Balaganesan G., Lee J.K.Y., Neisiany R.E., Surendran S., Ramakrishna S. (2019). A Review of Recent Advances in Nanoengineered Polymer Composites. Polymers.

[2] Benavides P.T., Lee U., Zarè-Mehrjerdi O. (2020). Life cycle greenhouse gas emissions and energy use of polylactic acid, bio-derived polyethylene, and fossil-derived polyethylene. J. Clean. Prod..

[3] Esameely Neisiany R., Enayati M.S., Sajkiewicz P., Pahlevanneshan Z., Ramakrishna S. (2020). Insight into the Current Directions in Functionalized Nanocomposite Hydrogels. Front. Mater..

[4] Foroughi F., Ghomi E.R., Dehaghi F.M., Borayek R., Ramakrishna S. (2021). A Review on the Life Cycle Assessment of Cellulose: From Properties to the Potential of Making It a Low Carbon Material. Materials.

[5] Ryan P.G. (2015). A Brief History of Marine Litter Research. Marine Anthropogenic Litter.

[6] Ramesh P., Vinodh S. (2020). State of art review on Life Cycle Assessment of polymers. Int. J. Sustain. Eng..

[7] Feghali E., Tauk L., Ortiz P., Vanbroekhoven K., Eevers W. (2020). Catalytic chemical recycling of biodegradable polyesters. Polym. Degrad. Stab..

[8] Suwanmanee U., Varabuntoonvit V., Chaiwutthinan P., Tajan M., Mungcharoen T., Leejarkpai T. (2012). Life cycle assessment of single use thermoform boxes made from polystyrene (PS), polylactic acid, (PLA), and PLA/starch: Cradle to consumer gate. Int. J. Life Cycle Assess..

[9] Rodríguez L.J., Fabbri S., Orrego C.E., Owsianiak M. (2020). Comparative life cycle assessment of coffee jar lids made from biocomposites containing poly (lactic acid) and banana fiber. J. Environ. Manag..

[10] Ghomi E.R., Khosravi F., Tahavori M.A., Ramakrishna S. (2021). Circular Economy: A Comparison Between the Case of Singapore and France. Mater. Circ. Econ..

[11] Neisiany R.E., Enayati M.S., Kazemi-Beydokhti A., Das O., Ramakrishna S. (2020). Multilayered Bio-Based Electrospun Membranes: A Potential Porous Media for Filtration Applications. Front. Mater..

[12] Benavides P.T., Dunn J.B., Han J., Biddy M., Markham J. (2018). Exploring Comparative Energy and Environmental Benefits of Virgin, Recycled, and Bio-Derived PET Bottles. ACS Sustain. Chem. Eng..

[13] Hottle T.A., Bilec M.M., Landis A.E. (2017). Biopolymer production and end of life comparisons using life cycle assessment. Resour. Conserv. Recycl..

[14] Bishop G., Styles D., Lens P.N. (2021). Environmental performance comparison of bioplastics and petrochemical plastics: A review of life cycle assessment (LCA) methodological decisions. Resour. Conserv. Recycl..

[15] Morão A., De Bie F. (2019). Life Cycle Impact Assessment of Polylactic Acid (PLA) Produced from Sugarcane in Thailand. J. Polym. Environ..

[16] Nitkiewicz T., Wojnarowska M., Sołtysik M., Kaczmarski A., Witko T., Ingrao C., Guzik M. (2020). How sustainable are biopolymers? Findings from a life cycle assessment of polyhydroxyalkanoate production from rapeseed-oil derivatives. Sci. Total. Environ..

[17] Choi B., Yoo S., Park S.-I. (2018). Carbon Footprint of Packaging Films Made from LDPE, PLA, and PLA/PBAT Blends in South Korea. Sustainability.

[18] Rojas-Bringas P.M., De-La-Torre G.E., Torres F.G. (2021). Influence of the source of starch and plasticizers on the environmental burden of starch-Brazil nut fiber biocomposite production: A life cycle assessment approach. Sci. Total. Environ..

[19] Haylock R., Rosentrater K.A. (2017). Cradle-to-Grave Life Cycle Assessment and Techno-Economic Analysis of Polylactic Acid Composites with Traditional and Bio-Based Fillers. J. Polym. Environ..

[20] Penadés-Plà V., Martí J.V., García-Segura T., Yepes V. (2017). Life-Cycle Assessment: A Comparison between Two Optimal Post-Tensioned Concrete Box-Girder Road Bridges. Sustainability.

[21] Walker S., Rothman R. (2020). Life cycle assessment of bio-based and fossil-based plastic: A review. J. Clean. Prod..

[22] Lorite G., Rocha J.M., Miilumäki N., Saavalainen P., Selkälä T., Morales-Cid G., Gonçalves M., Pongrácz E., Rocha C.M., Toth G. (2017). Evaluation of physicochemical/microbial properties and life cycle assessment (LCA) of PLA-based nanocomposite active packaging. LWT.

[23] Maga D., Hiebel M., Aryan V. (2019). A Comparative Life Cycle Assessment of Meat Trays Made of Various Packaging Materials. Sustainability.

[24] Chitaka T.Y., Russo V., Von Blottnitz H. (2020). In pursuit of environmentally friendly straws: A comparative life cycle assessment of five straw material options in South Africa. Int. J. Life Cycle Assess..

[25] Gregoriadis G. (1979). Drug Carriers in Biology and Medicine.

[26] Karamanlioglu M., Preziosi R., Robson G.D. (2017). Abiotic and biotic environmental degradation of the bioplastic polymer poly(lactic acid): A review. Polym. Degrad. Stab..

[27] Momeni S., Ghomi E.R., Shakiba M., Shafiei-Navid S., Abdouss M., Bigham A., Khosravi F., Ahmadi Z., Faraji M., Abdouss H. (2021). The Effect of Poly (Ethylene glycol) Emulation on the Degradation of PLA/Starch Composites. Polymers.

[28] Blanco I., Ingrao C., Siracusa V. (2020). Life-Cycle Assessment in the Polymeric Sector: A Comprehensive Review of Application Experiences on the Italian Scale. Polymers.

[29] Lambert S., Wagner M. (2017). Environmental performance of bio-based and biodegradable plastics: The road ahead. Chem. Soc. Rev..

[30] Correa-Pacheco Z.N., Black-Solís J.D., Ortega-Gudiño P., Sabino-Gutiérrez M.A., Benítez-Jiménez J.J., Barajas-Cervantes A., Bautista-Baños S., Hurtado-Colmenares L.B. (2019). Preparation and Characterization of Bio-Based PLA/PBAT and Cinnamon Essential Oil Polymer Fibers and Life-Cycle Assessment from Hydrolytic Degradation. Polymers.

[31] Aldas M., Pavon C., De La Rosa-Ramírez H., Ferri J.M., Bertomeu D., Samper M.D., López-Martínez J. (2021). The Impact of Biodegradable Plastics in the Properties of Recycled Polyethylene Terephthalate. J. Polym. Environ..

[32] Cakir S., Aycicek M., Akinci A. (2018). Investigation of the mechanical and physical properties of PLA produced by injection molding for matrix material of polymer composites. Mater. Sci. Adv. Compos. Mater..

[33] Farah S., Anderson D.G., Langer R. (2016). Physical and mechanical properties of PLA, and their functions in widespread applications—A comprehensive review. Adv. Drug Deliv. Rev..

[34] Luedtke J., Gaugler M., Grigsby W.J., Krause A. (2019). Understanding the development of interfacial bonding within PLA/wood-based thermoplastic sandwich composites. Ind. Crop. Prod..

[35] Karamanlioglu M., Alkan U. (2019). Influence of time and room temperature on mechanical and thermal degradation of poly(lactic) acid. Therm. Sci..

[36] Mazidi M.M., Edalat A., Berahman R., Hosseini F.S. (2018). Highly-Toughened Polylactide- (PLA-) Based Ternary Blends with Significantly Enhanced Glass Transition and Melt Strength: Tailoring the Interfacial Interactions, Phase Morphology, and Performance. Macromolecules.

[37] Wang X., Pan Y., Yuan H., Su M., Shao C., Liu C., Guo Z., Shen C., Liu X. (2020). Simple fabrication of superhydrophobic PLA with honeycomb-like structures for high-efficiency oil-water separation. Chin. Chem. Lett..

[38] Yuan Z., Zhang X., Yao Q., Zhang Y., Fu Y. (2019). Production of acetonitrile via catalytic fast pyrolysis of biomass derived polylactic acid under ammonia atmosphere. J. Anal. Appl. Pyrolysis.

[39] Pavezi K.J., Rocha A., Bonafé E.G., Martins A.F. (2020). Electrospinning-electrospraying of poly(acid lactic) solutions in binary chloroform/formic acid and chloroform/acetic acid mixtures. J. Mol. Liq..

[40] Södergård A., Stolt M. (2002). Properties of lactic acid based polymers and their correlation with composition. Prog. Polym. Sci..

[41] Jin F.-L., Hu R.-R., Park S.-J. (2019). Improvement of thermal behaviors of biodegradable poly(lactic acid) polymer: A review. Compos. Part B Eng..

[42] Wang M., Wu Y., Li Y.-D., Zeng J.-B. (2017). Progress in Toughening Poly(Lactic Acid) with Renewable Polymers. Polym. Rev..

[43] Tsuji H., Kondoh F. (2017). Synthesis of meso-lactide by thermal configurational inversion and depolymerization of poly(l-lactide) and thermal configurational inversion of lactides. Polym. Degrad. Stab..

[44] Scaffaro R., Botta L., Maio A., Gallo G. (2017). PLA graphene nanoplatelets nanocomposites: Physical properties and release kinetics of an antimicrobial agent. Compos. Part B Eng..

[45] Chen C.-C., Chueh J.-Y., Tseng H., Huang H.-M., Lee S.-Y. (2003). Preparation and characterization of biodegradable PLA polymeric blends. Biomaterials.

[46] Lim J.S., Park K.-I., Chung G.S., Kim J.H. (2013). Effect of composition ratio on the thermal and physical properties of semicrystalline PLA/PHB-HHx composites. Mater. Sci. Eng. C.

[47] Sedničková M., Pekařová S., Kucharczyk P., Bočkaj J., Janigová I., Kleinová A., Jochec-Mošková D., Omaníková L., Perďochová D., Koutný M. (2018). Changes of physical properties of PLA-based blends during early stage of biodegradation in compost. Int. J. Biol. Macromol..

[48] Ochi S. (2008). Mechanical properties of kenaf fibers and kenaf/PLA composites. Mech. Mater..

[49] Mathew A.P., Oksman K., Sain M. (2005). Mechanical properties of biodegradable composites from poly lactic acid (PLA) and microcrystalline cellulose (MCC). J. Appl. Polym. Sci..

[50] Yang S.-L., Wu Z.-H., Yang W., Yang M.-B. (2008). Thermal and mechanical properties of chemical crosslinked polylactide (PLA). Polym. Test..

[51] Suryanegara L., Nakagaito A.N., Yano H. (2009). The effect of crystallization of PLA on the thermal and mechanical properties of microfibrillated cellulose-reinforced PLA composites. Compos. Sci. Technol..

[52] Castro-Aguirre E., Iñiguez-Franco F., Samsudin H., Fang X., Auras R. (2016). Poly(lactic acid)—Mass production, processing, industrial applications, and end of life. Adv. Drug Deliv. Rev..

[53] Keoleian G., Miller S., Kleine R., Fang A., Mosley J. (2012). Life Cycle Material Data Update for GREET Model.

[54] Vink E.T., Davies S. (2015). Life Cycle Inventory and Impact Assessment Data for 2014 Ingeo™ Polylactide Production. Ind. Biotechnol..

[55] Zaaba N.F., Jaafar M. (2020). A review on degradation mechanisms of polylactic acid: Hydrolytic, photodegradative, microbial, and enzymatic degradation. Polym. Eng. Sci..

[56] Muller J., González-Martínez C., Chiralt A. (2017). Combination of Poly(lactic) Acid and Starch for Biodegradable Food Packaging. Materials.

[57] Roy S., Rhim J.-W. (2020). Preparation of bioactive functional poly(lactic acid)/curcumin composite film for food packaging application. Int. J. Biol. Macromol..

[58] Bałdowska-Witos P., Piotrowska K., Kruszelnicka W., Błaszczak M., Tomporowski A., Opielak M., Kasner R., Flizikowski J. (2020). Managing the Uncertainty and Accuracy of Life Cycle Assessment Results for the Process of Beverage Bottle Moulding. Polymers.

[59] Khosravi A., Fereidoon A., Khorasani M.M., Naderi G., Ganjali M.R., Zarrintaj P., Saeb M.R., Gutiérrez T.J. (2020). Soft and hard sections from cellulose-reinforced poly(lactic acid)-based food packaging films: A critical review. Food Packag. Shelf Life.

[60] Gan I., Chow W. (2018). Antimicrobial poly(lactic acid)/cellulose bionanocomposite for food packaging application: A review. Food Packag. Shelf Life.

[61] Lasprilla A.J., Martinez G.A., Lunelli B.H., Jardini A.L., Filho R.M. (2012). Poly-lactic acid synthesis for application in biomedical devices—A review. Biotechnol. Adv..

[62] Pawar R.P., U Tekale S., U Shisodia S., T Totre J., Domb A.J. (2014). Biomedical applications of poly (lactic acid). Recent Pat. Regen. Med..

[63] Rezvani Ghomi E., Khosravi F., Neisiany R.E., Singh S., Ramakrishna S. (2021). Future of additive manufacturing in healthcare. Curr. Opin. Biomed. Eng..

[64] Eshkalak S.K., Ghomi E.R., Dai Y., Choudhury D., Ramakrishna S. (2020). The role of three-dimensional printing in healthcare and medicine. Mater. Des..

[65] Zhao P., Rao C., Gu F., Sharmin N., Fu J. (2018). Close-looped recycling of polylactic acid used in 3D printing: An experimental investigation and life cycle assessment. J. Clean. Prod..

[66] Rossi V., Cleeve-Edwards N., Lundquist L., Schenker U., Dubois C., Humbert S., Jolliet O. (2015). Life cycle assessment of end-of-life options for two biodegradable packaging materials: Sound application of the European waste hierarchy. J. Clean. Prod..

[67] Wan L., Zhang Y. (2018). Jointly modified mechanical properties and accelerated hydrolytic degradation of PLA by interface reinforcement of PLA-WF. J. Mech. Behav. Biomed. Mater..

[68] Chamas A., Moon H., Zheng J., Qiu Y., Tabassum T., Jang J.H., Abu-Omar M.M., Scott S.L., Suh S. (2020). Degradation Rates of Plastics in the Environment. ACS Sustain. Chem. Eng..

[69] De Andrade M.F.C., Souza P.M.S., Cavalett O., Morales A.R. (2016). Life Cycle Assessment of Poly(Lactic Acid) (PLA): Comparison Between Chemical Recycling, Mechanical Recycling and Composting. J. Polym. Environ..

[70] Maga D., Hiebel M., Thonemann N. (2019). Life cycle assessment of recycling options for polylactic acid. Resour. Conserv. Recycl..

[71] Mallet B., Lamnawar K., Maazouz A. (2013). Improvement of blown film extrusion of poly(Lactic Acid): Structure-Processing-Properties relationships. Polym. Eng. Sci..

[72] Jaszkiewicz A., Bledzki A.K., Van Der Meer R., Franciszczak P., Meljon A. (2014). How does a chain-extended polylactide behave?: A comprehensive analysis of the material, structural and mechanical properties. Polym. Bull..

[73] Meng Q.-K., Heuzey M.-C., Carreau P.J. (2012). Effects of a Multifunctional Polymeric Chain Extender on the Properties of Polylactide and Polylactide/Clay Nanocomposites. Int. Polym. Process..

[74] Lyu S., Schley J., Loy B., Lind D., Hobot C., Sparer R., Untereker D. (2007). Kinetics and Time−Temperature Equivalence of Polymer Degradation. Biomacromolecules.

[75] Vink E.T., Rábago K.R., Glassner D.A., Gruber P.R. (2003). Applications of life cycle assessment to NatureWorks™ polylactide (PLA) production. Polym. Degrad. Stab..

[76] Madival S., Auras R., Singh S.P., Narayan R. (2009). Assessment of the environmental profile of PLA, PET and PS clamshell containers using LCA methodology. J. Clean. Prod..

[77] Piemonte V. (2011). Bioplastic Wastes: The Best Final Disposition for Energy Saving. J. Polym. Environ..

[78] Papong S., Malakul P., Trungkavashirakun R., Wenunun P., Chom-In T., Nithitanakul M., Sarobol E. (2014). Comparative assessment of the environmental profile of PLA and PET drinking water bottles from a life cycle perspective. J. Clean. Prod..

[79] Horowitz N., Frago J., Mu D. (2018). Life cycle assessment of bottled water: A case study of Green2O products. Waste Manag..

[80] Mahalle L., Alemdar A., Mihai M., Legros N. (2014). A cradle-to-gate life cycle assessment of wood fibre-reinforced polylactic acid (PLA) and polylactic acid/thermoplastic starch (PLA/TPS) biocomposites. Int. J. Life Cycle Assess..

[81] Benetto E., Jury C., Igos E., Carton J., Hild P., Vergne C., Di Martino J. (2015). Using atmospheric plasma to design multilayer film from polylactic acid and thermoplastic starch: A screening Life Cycle Assessment. J. Clean. Prod..

[82] Bałdowska-Witos P., Kruszelnicka W., Kasner R., Tomporowski A., Flizikowski J., Kłos Z., Piotrowska K., Markowska K. (2020). Application of LCA Method for Assessment of Environmental Impacts of a Polylactide (PLA) Bottle Shaping. Polymers.

[83] Molins G., Álvarez M.D., Garrido N., Macanás J., Carrillo F. (2018). Environmental Impact Assessment of Polylactide(PLA)/Chicken Feathers Biocomposite Materials. J. Polym. Environ..

[84] Carus M. (2017). Biobased Economy and Climate Change—Important Links, Pitfalls, and Opportunities. Ind. Biotechnol..

